# The Antioxidant Effect of the Metal and Metal-Oxide Nanoparticles

**DOI:** 10.3390/antiox11040791

**Published:** 2022-04-18

**Authors:** Xuemei Ge, Zhaoxin Cao, Lanling Chu

**Affiliations:** Department of Food Science and Technology, College of Light Industry Science and Engineering, Nanjing Forestry University, 159 Longpan Road, Nanjing 210037, China; gexuemei@njfu.edu.cn (X.G.); caozhaoxin1225@163.com (Z.C.)

**Keywords:** inorganic nanoparticles, antioxidant, applications, mechanism, toxicity

## Abstract

Inorganic nanoparticles, such as CeO_3_, TiO_2_ and Fe_3_O_4_ could be served as a platform for their excellent performance in antioxidant effect. They may offer the feasibility to be further developed for their smaller and controllable sizes, flexibility to be modified, relative low toxicity as well as ease of preparation. In this work, the recent progress of these nanoparticles were illustrated, and the antioxidant mechanism of the inorganic nanoparticles were introduced, which mainly included antioxidant enzyme-mimetic activity and antioxidant ROS/RNS scavenging activity. The antioxidant effects and the applications of several nanoparticles, such as CeO_3_, Fe_3_O_4_, TiO_2_ and Se, are summarized in this paper. The potential toxicity of these nanoparticles both in vitro and in vivo was well studied for the further applications. Future directions of how to utilize these inorganic nanoparticles to be further applied in some fields, such as medicine, cosmetic and functional food additives were also investigated in this paper.

## 1. Introduction

Nanotechnology functions as a platform to bridge the materials from the molecular and atomic levels to bulk, which has been widely developed in many fields such as energy, environment protection, and healthcare [1]. The preparation of the nanoparticles could be mainly divided into two different strategies: bottom-up and top-down [2]. Also, the system engineering was developed to scale up the nanoparticle manufacturing from bench to industry for their further applications [3]. Depending on the forming materials, these nanoparticles could be mainly classified into organic polymer formed nanoparticles and the inorganic nanoparticles such as metal oxidants nanoparticles. Among them, most of the polymers (including a natural polymer and synthetic polymers) formed nanoparticles could be applied as carriers to encapsulate bioactive agents for the purpose of improving their stability and efficacy. The inorganic nanoparticle was recognized as nano-scaled particles to be applied as carriers or exhibit bioactivities, which have drawn much attention to be applied in fields such as medicine, cosmetics, agriculture, functional foods development and packaging [4,5,6,7,8,9]. More research was performed regarding the inorganic particles due to their good performance, such as physical, chemical, mechanical stability, compatibility with other compounds (e.g., synthetic polymers) and whether they were easy to prepare or modify [10,11,12]. 

Oxidant is recognized as one class of the factor for series of diseases and ageing [13,14,15,16,17]. Reactive oxygen species (ROS) are commonly produced by the natural oxidative process, which is formed by reduction–oxidation reactions or by electronic excitation [18]. Evidence suggested that aging may relate to reactive oxygen species (ROS) damage. The popular theory regarding aging in recent years was the free radical theory of aging, focusing on mitochondria as a source as well as a target of ROS [14,19,20]. The recent research was mostly focused on how to reduce these oxidant effects. The nano-scaled inorganic particles could be well developed for their further application of antioxidant effects in many fields, such as medicine, cosmetic and functional foods additives. The smaller sizes of inorganic nanoparticles could offer more feasibility to function as antioxidants due to the high surface to volume ratio. Although metallic and metallic oxide nanoparticles were considered to potentially induce oxidative stress and lead to undesirable health problems, some of the nanoparticles exhibit excellent behavior in antioxidants, such as TiO_2_, cerium oxide nanoparticles and Fe_3_O_4_ in recent research works [21]. TiO_2_ were widely used in many products due to their low toxicity, and has to be considered as a nano-material on cellular antioxidant defense [22]. Also, cerium oxide nanoparticles may function as a free radical scavenger regeneratively to defect oxygen with their lattice structure [23]. Fe_3_O_4_ could be further modified to carbon paste electrode to form nanoparticles for electrosensitive determination of antioxidant components, such as sinapic acid, syringic acid and rutin [24]. 

The preparation of different types of inorganic nanoparticles which could offer the feasibility of antioxidant effects is described in this paper. The different characteristics of the inorganic nanoparticles, such as morphology, sizes, zeta potential agglomeration and magnetization, could affect their behavior. Depending on the requirements of application, production of inorganic nanoparticles is usually variable, such as laser ablation, microwave synthesis and electrodeposition [25,26,27]. This paper summarizes recent advances in fabrication of inorganic nanoparticles. The mechanism of their antioxidant effects is also introduced, which mainly includes antioxidant enzyme-mimic effect and antioxidant ROS/RNS scavenging activity [28,29,30]. Some typical inorganic nanoparticles, such as TiO_2_, cerium oxidant nanoparticles and Fe_3_O_4_ with excellent performance in oxidant effects are introduced as well. Although nanoparticles have attracted widespread attention in many fields, the questions on their potential toxicity, safety and metabolization after application still need to be addressed. Also, the behavior of these inorganic nanoparticles with the different types, such as sizes, zeta potential and sharps, requires further investigation after administration. 

## 2. Preparation of Different Inorganic Nanoparticles

The preparation methods of inorganic nanoparticles are variable, and most were depended on the purpose of their applications. Generally, it consists of physical, chemical and biological processes [31,32,33]. The conventional techniques involved in the preparation of the inorganic nanoparticles mainly included electrodeposition, sol-gel synthesis and thermal decomposition methods [34]. The sol-gel methods, which refer to mixing compounds containing highly active liquid phases, could dramatically reduce the costs of manufacturing, produce pure inorganic nanoparticles and lower the wastes such as solvent [35,36]. Electrodeposition could be used as a strategy to fabricate inorganic nanoparticles, by which the precursor, such as dissolved Fe^2+^ or Fe^3+^ ions, can be deposited to form the nano-scaled particulates [37,38]. The thermal decomposition method was processed by setting external controlling conditions, and the insoluble substances are dissolved under high-temperature or high-pressure conditions. After recrystallizing, the nanoparticle was obtained by proper separation method. This method allows for simple fabrication of nanoparticles, with low cost and pollution [39,40]. 

**Figure 1:**
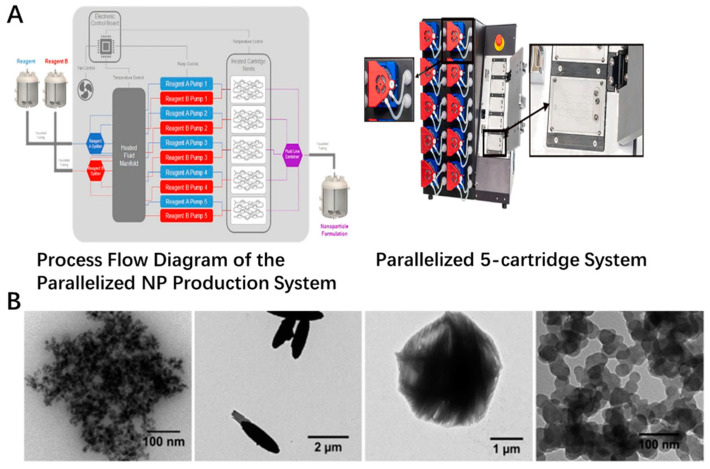
Scale-up platform currently in development and inorganic particle species formed on-chip. (**A**) Flow schematic of high-throughput assembly. The structure of microfluid platform with insets of disposable positive displacement pumps and cartridge holder that does not require manual fluidic connections. (**B**) TEM images of ZnO particles prepared by using LGD variant at FRR of 1:1, 1:6, and 1:20. Iron oxide particles fabricated at a TFR of 10.5 mL/min and FRR of 1:2.

The newer method was also incorporated to form the inorganic nanoparticles recently, which could provide more feasibility for low cost, high production, uniform sizes and scale up for industrial applications. Newer techniques, such as laser ablation and microwave synthesis, could be applied in the fabrication of the inorganic nanoparticles [41,42,43,44,45]. Microwaves could offer the feasibility to assist the synthesis process, which was explored in the producing of inorganic nanomaterials. The CeO_2_ was well prepared by the microwave heating method. The crystal size of these nanoparticles was around 8 nm in a sphere-like shape [46]. The green synthesis was also used for the fabrication of these metal oxide nanoparticles. One example is the synthesis of superparamagnetic Fe_3_O_4_ nanoparticles using α-glucose as the reducing agent. The formed nanoparticles were roughly spherical in shape, with a size of around 12.5 nm [47]. TiO_2_ nanoparticles could be also prepared with mango peel extracted under acidic conditions by green synthesis concept. The crystal structure, size and crystallinity vary by the different ratios of the extract to TiCl_3_, which was decreased with increasing the extract content [48]. Vitamin C could be used for the green synthesis of cluster-grapes nanoparticles. The mechanism was a plausible reaction with a two-electron transfer pathway of acid-inhibition nature [49]. These works could offer a mild, environmentally friendly and economical strategy for the preparation of these metal oxide nanoparticles. Also, the use of a microfluidics method, especially the combination of new technologies, could open new possibilities for this field. As shown in Figure 1A, different reagent inputs enter the fluidic network and are heated to the desired temperature, after which the streams are split and delivered to all five cartridges. Particles are formed on chip and pooled. For example, zinc oxide particles could be prepared by microfluidic device simply by mixing zinc nitrate solution with NaOH. The final size of the micro or nano-particulates could be controlled through the concentration of metal precursor, solvent and the total flow rates. At low reagent concentrations with low FRR, such as 1:1, the total surface energy could be decreased to form spherical ZnO particles. With the increase of the FRR to 1:6, the ellipsoid particle formed. When FRR reached 1:20, the urchin-shaped particle could be generated. The iron oxide nanoparticles could also be fabricated in this method with uniform sizes of around 36 nm in spherical shape (Figure 1B). All operations are controlled from an intuitive software interface, which allows them to be easily applied to consumers. The scale-up compatibility of this microfluidic platform carried out to ensure the batch to batch reproducibility, and in-line monitoring of the particle size with the absorbance in real time by commercial products was also coupled to ensure the quality of the particulates during the producing process(as shown in Figure 1) [50]. 

Additionally, the surface modification of the inorganic nanoparticles was also incorporated into the fabricating of inorganic nanoparticles [7,11]. It could optimize the diversity of inorganic nanoparticles in size, shape, solubility and long-term stability. Also, the modification could offer the feasibility of these nanoparticles to further immobilize the functional groups as well as change the behavior after applied. The surface modification of the nanoparticle could be achieved by strategies such as chemical modification, self-assembly method, emulsion method or other related techniques [51,52,53]. These strategies included grafting the moieties on the nanoparticle surface, exchanging the ligands on the surface of the nanoparticles or chemical conjugating of the functional groups. Modification of the metal oxide nanoparticle, such as carboxylate, silanes and phosphonates, could be performed to achieve the functional surfaces [54]. Also, NaOH and gluconic acid were used as solvents and surfactants for the purpose of synthesis surface modified codoped zinc oxide. The surface modification could affect the shape and size distribution of the ZnO nanoparticles as well as their scavenging abilities [55]. 

## 3. Mechanism of Antioxidant Effects

### 3.1. Antioxidant Effects of the Modified Nanoparticles

The modification of the inorganic nanoparticles could immobilize some functional groups, which could provide antioxidant activity. The nanoparticle could be modified by chemical reaction or by some other method, such as the self-assembly method [56,57,58]. One example is that the 3-(3,5-di-tert-butyl-4-hydroxyphenyl) propionic acid (DPPH), which is one kind of phenol antioxidant organic compound, could be modified on the surface of the ZnO to scavenge the radicals. The linkage could be formed between DBHP and ZnO nanoparticles [59]. Also, the DBHP-ZnO nanoparticles could improve the efficiency of DBHP in scavenging the radicals produced by the oil oxidation process [60]. Convent coupling of the antioxidant functional moieties or entrapping the functional bioactivates on the surface of the inorganic nanoparticles could serve as one of the fantastic strategies to combine the surface activities of the nanoscaled particles together with the antioxidant effects of incorporated functional moieties [61,62,63]. The nano-formulated quercetin was loaded in calcium phosphate nanoparticles, which could exhibit pH indicator, fluorophore and antioxidant effects. The pretreatment of these formulations could protect the cells from H_2_O_2_-mediated toxicity [61]. Also, the antioxidant functional substance, which could help to form the inorganic nanoparticles, such as some natural fruit extract, could also be absorbed on the surface of the nanoparticulate to scavenge the free radical for its antioxidant effects. Gold nanoparticles were fabricated by ripened Capuli (*Prunus serotina* Ehrh. var. Capuli) fruit-derived extracts in an ecofriendly way. The formed nanoparticles were spherical and triangular in shape with the size ranging from 30 to 400 nm. These nanoparticles could exhibit a 46.12% inhibition percentage of DPPH for 30 min, which was mostly attributed to the adsorption/binding of the extract phytocompounds on the surface of the gold nanoparticles [64]. Some other metal oxide nanoparticles, such as Fe_2_O_3_, TiO_2_ and CuO, also have the antioxidant effect due to the incorporation of natural extracts from plants or fruit on their surface, which could provide the bioactive function [65].

### 3.2. Antioxidant Enzyme-Mimetic Activity

Oxidative stress is the main factor which could induce related diseases associated with the imbalanced ROS and antioxidant defenses such as the free radical cleavage substances. Recently, the research works involved in the inorganic nanoparticles in antioxidant effects have explained the mechanism of their activities. The effects of the antioxidant could be explained as an antioxidant enzyme-mimetic mechanism. For example, the superoxide dismutase(SOD) mimetics could function as catalytic agents to remove superoxide and peroxynitrite [66]. Korsvik et al. first revealed that Ce Nanoparticles (CNP_S_) could exhibit the effects of the SOD enzyme mimetic activities [67]. They speculate that the mechanism of the CNP_S_ to the O_2_^−^ was catalyzed as Equation (1) as below:

O_2_^−^ + Ce^4+^ → O_2_ + Ce^3+^                    O_2_^−^ + Ce^3+^ + 2H^+^ → H_2_O_2_ + Ce^4+^ (1)

It is obvious that the Ce^3+^/Ce^4+^ could regenerate during their functions. Also, nanoparticles could offer more possibility to increase the reactivity due to the large surface to volume ratio [68]. The ceria nanoparticles with higher Ce^3+^/Ce^4+^ could achieve SOD mimetic activity which was assayed using ferricytochrome C [67]. The Ce oxidant nanoparticles could have catalase mimetic activity on the H_2_O_2_, which was much more damaging due to the inducing of OH. Most of their effects depended on the ratio of the Ce^3+^ to Ce^4+^, as well as other parameters, such as the preparation procedure [69]. Additionally, the CNPs could produce a series of enzyme-like activities, such as phosphatase mimetic [70], oxidase mimetic [71], peroxidase mimetic [72] and ATPase mimetic effects [73], and allow them to be applied in broad fields, such as medicine, functional additives and environmental sciences [74,75]. 

Also, glutathione peroxidase (GPx)-like enzymes are known to affect the H_2_O_2_ level intra- and intercellularly, which involves glutathione(GSH) as a co-factor. The V_2_O_5_ nanowires could exhibit the GPx enzymes activity in presence of GSH, thus prohibiting the processing of the cell oxidant damage. The variations in the GPx enzyme-like activity may be attributed to the difference in the rate of the V-peroxide species formation on the surface of the nanostructure [76]. 

### 3.3. Antioxidant ROS/RNS Scavenging Activity

Reactive oxygen species (ROS) are usually generated by breaking covalent bonds of molecules during natural oxidative processes [77]. Formation of the different ROS, which included molecules derived from molecular oxygen by reduction-oxidation reaction, as well as electronic excitation, could induce the molecular damage [17]. ROS are generated by various sources both endogenous and exogenous. For example, H_2_O_2_ was one of the major ROS-induced substances and could maintain its effects even at low nano-molar under stimulated stress, e.g., growth factors, chemokines or other stressors. Some of the inorganic nanoparticles could exhibit their effects by enzyme mimetic behavior, such as SOD-mimetic activity, to function as reducing agents. They could also scavenge the ROS effectively. It has been suggested that the CNPs could remove the OH produced by H_2_O_2_, most of which were also based on the ratio of the Ce^3+^ [78]. The switch from Ce^3+^ to Ce^4+^ could well explain the scavenging activity of the antioxidant inorganic nanoparticle in ROS. Also, the CNPs were considered as activities in reducing the nitrosative stress, such as NO and O_2_NO^−^ [79,80,81]. In addition to Ce oxidant nanoparticles, other inorganic nanoparticles could also have the efficiency in scavenging of the ROS/RNS. Some of the effects were due to the special electronic configuration such as La element-based oxidant nanoparticle. The surface modification could be recognized as one of the strategies to couple with functional moieties or coating on the nanoparticles for the purpose of exhibiting antioxidant effects. Also, selenium could function as the bioactive element to inhibit the damage of ROS scavenging. The selenium nanoparticles could scavenge the free radicals, and most of the selenium could participate in the activities of important antioxidant enzymes [82]. 

## 4. The Antioxidant Effects of Different NPs

### 4.1. Ce Oxide Nanoparticle

Cerium is one kind of rare earth element in the lanthanide series of elements and could exhibit a high tolerance for reversible oxidation/reduction due to the oxidation state cerium and cerium cycling [83]. As one of the metal-based inorganic nanoparticles, cerium oxide particulates could offer the excellent behavior in antioxidant effects. Compared with bulk forms of cerium, the nanoparticle has a much higher catalytic effect, which was mostly attributed to the increased surface to volume ratio [84]. The mechanism of the antioxidant effect of the cerium nanoparticles were mainly divided into the antioxidant enzyme mimetic activity and the antioxidant ROS/RNS scavenging activity. The mechanism regarding the antioxidant enzyme mimetic mechanism could be well explained as the chemical reaction equation as shown in (1). In addition to SOD enzyme mimetic and CAT enzyme mimetic activities, the cerium could also have phosphatase-like, oxidase-like, peroxidase-like as well as ATPase-like mimetic activity, which was attributed to other related functions [72,85,86,87,88]. For the mechanism of ROS/RNS scavenging antioxidant activity, the nanoparticle could be effective in reducing the free radicals, such as ROS or RNS. They could be potentially applied to the quenching of hydroxyl radicals, superoxide, peroxide and as well as nitric oxide [67,89,90]. The mechanism of the cerium to function as enzyme-like activities or ROS/RNS scavenging activities was described in Table 1. 

**Figure 2:**
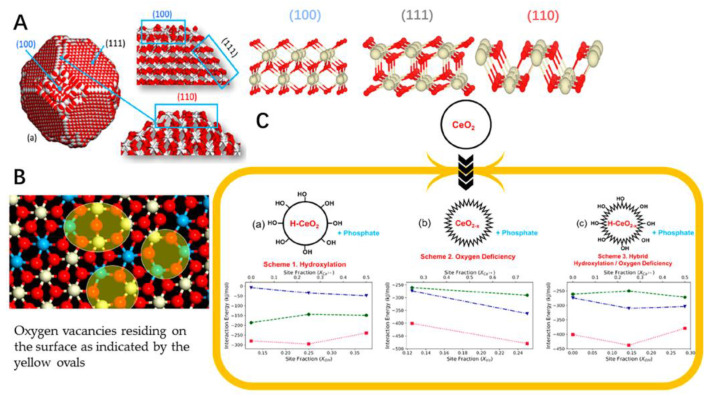
(**A**) Structure of a nanoparticle of ceria showing 111, 110 and 100 surfaces. The structures of “perfect” 111, 110 and 100 surfaces of nanoceria simulated using DFT are consistent with the structures of the surfaces exposed by the nanoparticle. (**B**) One of the nanoparticle’s 111 surfaces after nanoceria has been reduced. Ce^4+^ is in white, Ce^3+^ is in blue and oxygen is red. (**C**) Interaction energy of phosphate with nanoceria for three compositions of nanoceria in a living cell. Interaction energy (kJ/mol) of phosphate at nanoceria surfaces 111 (blue triangles), 110 (green circles) and 100 (red squares).

It has been proven that the cerium oxide nanoparticles could reduce oxidant-mediated apoptosis in target cells [97]. It has been demonstrated that cerium nanoparticles could have potential to be applied in treatment of oxidative-stress-related disease both in vitro and in vivo [98,99,100]. Also, some surface modification strategies of nano-scaled particulates could be used in decoration of the cerium nanoparticle, such as PEGylation. The cerium oxide nanoparticles have been shown to have low distribution in organs, which was less than 2% [101]. Physical adsorption and chemical covalent conjugation of cerium oxide nanoparticles and PEG could be the methods to graft the PEG on the surface of the nanoparticle to enhance the tissue accumulation [102,103]. The antioxidant effects and cytocompatibility of the different shape cerium oxide nanoparticles in rod- and cube-shaped was also studied. The PEGylation of the cerium oxide nanoparticles could reduce protein adsorption but did not induce the cytocompatibility. The rod-shaped cerium oxide nanoparticles could offer superior ROS scavenging activities to cube-shaped cerium oxide nanoparticles [102]. The modification of the cerium oxide nanoparticles could offer the desired behavior to be applied. Also, the computer aided design of the cerium oxide nanoparticles, which could be designed with different structures for the nanozyme-like activities (Figure 2). The model nanoparticle of CeO_2_ comprising 18,849 atoms was set up with three different structures involved in exposing 111, 110 and 100 surfaces. The presence of oxygen vacancies was revealed throughout the nanoparticles as well as on the surface. The interaction energy of phosphate with nanoceria for these compositions in living cells was also performed to determine the interaction strength (Figure 2C). The phosphate could directly interact strongly with the surface oxygen vacancies with the structure of OH^−^ and Ce^3+^ on hydroxylated oxygen-deficient surfaces [104]. These nanoparticles could be further prepared in different formulations for the purpose of the desired application in broad fields [105]. 

### 4.2. Fe Oxide Nanoparticle

**Figure 3:**
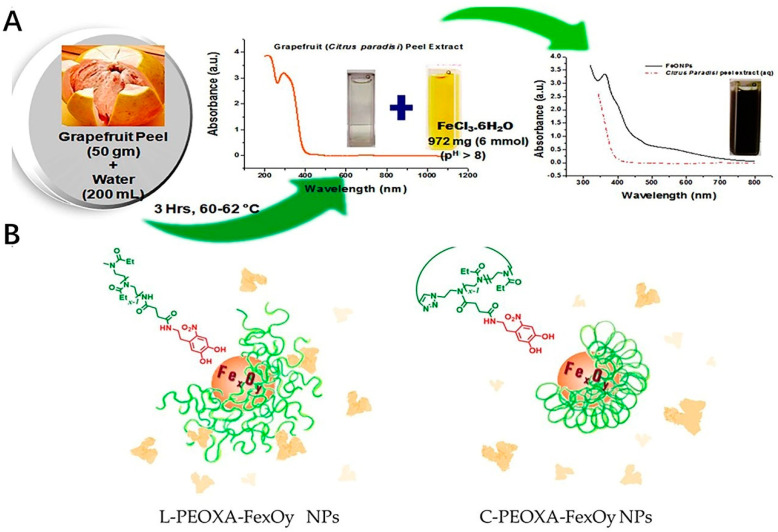
Examples of the fabrication process of the iron oxide nanoparticles. (**A**) Schematic representation of biosynthesis of FeONPs. (**B**) Surface modification of the nanoparticles. L−PEOXA−Fe_x_O_y_ and C−PEOXA−Fe_x_O_y_ NPs show different stability in HSA. Cyclic PEOXA shells could quantitatively prevent the formation of a protein corona. Linear brush shells cannot be entirely prevented.

The iron oxide nanoparticles have drawn much attention in modern nanotechnology due to their application in broad fields, such as biomedicine and environmental remediation [106]. The Fe oxide nanoparticles could be also modified to be applied as one of the inorganic nanoparticles for the purpose of the antioxidant efficacy. The effect of these particulates could perform well in the scavenging of the oxidative, most of which were fabricated with the precursors, such as some extracts from fruit or leaves [107]. Recently, green methods could be used for fabrication of the production of highly pure nanoparticles in an environmentally friendly way. This method involves using plant or fruit extracts and bio-organisms for the purpose of catalyzing the synthesis of nanoparticles [108,109]. The Fe oxide nanoparticles were fabricated by the *Phoenix dactylifera* L., one of the extracts from the plant leaves with high polyphenol content, which could exhibit the antioxidant activities by TAC (with the total antioxidant activity of 180 mg/g) as well as DPPH assay(with the IC_50_ DPPH value of 2.0 mg/mL) [110]. The nanoparticles of the iron oxide prepared by the green synthesis method of coriandrum sativum L. leaf activities could also have excellent free radical inhibitory activity [111]. Also, the iron oxide nanoparticles could be fabricated by the biological polyglucose-sorbitol-carboxymethyl ether (PSC) as the precursor in an environmental co-friendly way. The formed nanoparticles could have the ability to scavenge the ROS [112]. Mostly, the iron oxide nanoparticle, which could offer the antioxidant effect was fabricated together with the natural antioxidant activities by the green biosynthesis method, such as the leaf of Camellia sinensis and Zadirachta, fruit of Terminalia chebula and Passiflora tripartite, peel of Punica Cranatum and Pisum sativum, bran of Sorghums, seed of Syzygium cumimi, flower of Avicennia marina and Hibiscus sabdariffa, root of biomass of Medicago sativa [113]. As a sustainable approach, it could provide the strategy for the preparing of these metal nanoparticles easily as well as in an environmentally friendly way (Figure 3A). Also, the surface modification of these nanoparticles could improve their stability especially in protein-rich media. Compared with linear poly(2-ethyl-2-oxazoline) (PEOXA), cyclic PEOXA could offer a greater ability to prevent the formation of the protein corona around the nanoparticles as well as aggregation in the presence of HSA (Figure 3B). This is probably due to the cyclic PEOXA, which could form brush shells to hinder the surface. 

### 4.3. Titanium Oxide Nanoparticle

Titanium dioxide nanoparticles are potentially to be applied in food industry, as well as in other fields such as paper, plastics, paints and pharmaceuticals [114]. The titanium easily and rapidly oxidizes in air [115]. The titanium oxide nanoparticle was widely applied in irradiation of the UV-induced oxidant due to the creation of the barrier against ultraviolet rays, which allows them to be used as additives in cosmetic or sunscreen products. These nanoparticles could be potentially applied in broad fields, including their antioxidant effects in the plants such as Spinacia oleracea, Zea mays Cicer arietinum and Raphanus sativus [116]. It could increase antioxidant enzyme activity and decrease in malondialdehyde accumulation [117]. Also, the titanium dioxide nanoparticles could function as the carrier for the purpose of delivering antioxidants, such as quercetin, to improve their efficacy. The surface of the nanoparticle was modified with PEG, and these titanium nanoparticulates could yield better biocompatibility with uniform dispersion. The nanoparticle could also upregulate the phase 2 enzyme, which has antioxidative effects on the cells to reduce the oxidative toxicity [114]. However, the titanium dioxide nanoparticles could reduce the bioavailability of polyphenols due to the formation of large titanium dioxide nanoparticle-polyphenol complex agglomerates. Also, the binding could reduce the antioxidant effects of the polyphenol with low bioactivity [118]. The green synthesis with eco-friendly perspectives could be also applied in fabrication of titanium nanoparticles. The titanium nanoparticles could be biosynthesis by Morinda citrifolia, fruit waste of orange, peel extracts of the pomegranate, cassava, yam and lemon, the flower of caltotripis gigantean and cynodon dactylon. The Cuminum cyminus extracts could be used for the synthesis of titanium nanoparticles, which could also have good antioxidant potential by DPPH assay [114]. Also, this method could provide the method for the preparing these nanoparticles with low pollution, ease of operation as well as the application in industrial fields. Combined with surface modification, the antioxidant could be improved [114]. Although all of these could offer the feasibility of these nanoparticles to be applied as the actives, the potential bioavailibity as well as their stress on the environment should be addressed. 

### 4.4. Se

**Figure 4:**
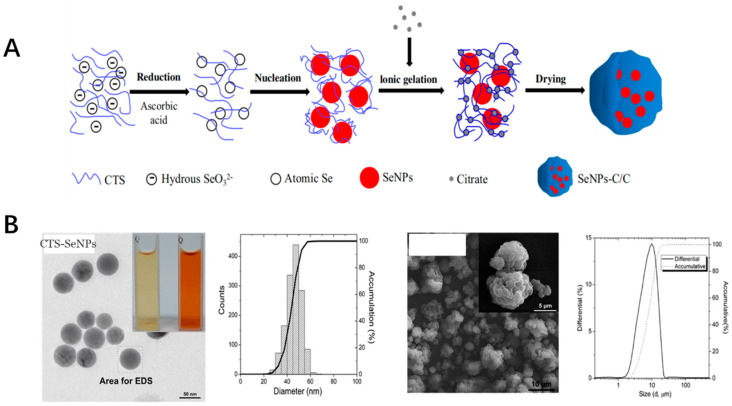
(**A**) The preparation process of SeNPs-C/C. (**B**) The SEM image and distribution of CTS-SeNPs as well as SeNPs-C/C.) [126].

Selenium is one of the trace elements for the dietary nutrients of the animals as well as human beings [119]. These nanoparticles were applied in widely fields such as functional food, agriculture(e.g. fish food) as well as the bioactive in pharmaceutics [120,121]. Selenium nanoparticles function as the special supplement of selenium due to their favorable higher bioactivities as antioxidant inorganic nanoparticles compared with other compound forms, such as sodium selenite, selenomethionine and methylselenocysteine [122]. Most of the selenium nanoparticles bioactivities were due to their participation in the important enzymes of the antioxidant defense system, such as glutathione [123]. The selenium nanoparticles could scavenge the free radicals with relatively low toxicity compared with other forms of the selenium compounds [124]. Selenium nanoparticles could be synthesized by physical, chemical, as well as the biological methods. Most research works were focused on developing the easy, economic and eco-friendly ways for fabricating of the selenium nanoparticles. The antioxidant activity was associated with the nanoparticle sizes and the stability [125]. The polysaccharides, monosaccharides, proteins, amino acids, polyphenols, melatonin, ATP, natural products extract as well as microorganism culture were applied to improve the stability of the selenium nanoparticles. One example is that the selenium nanoparticle was prepared together with chitosan, which was embedded into the microsphere by a spray-drying method. CTS and Vc were dissolved in an acetic acid solution. The aqueous selenite was added to the CTS/Vc solution slowly to obtain atomic Se. Atomic Se nucleated to form the Se nucleus, then were assembled into SeNPs. After that, mixed aqueous CTS and SeNPs were added to citrate solution to formulate SeNPs-C/C and then spray-dried to obtain solid SeNPs-C/C microparticles (Figure 4). Also, the toxicity of the prepared selenium nanoparticles was only 4-fold to 11-fold of that of selenite-based selenium dose with the same efficacy in increasing the GSH-Px activities as well as to reduce oxidative stress. The formed nanoparticles was with the uniformed diameter with excellent stability and acceptable release [126,127]. Some biological pathways could also be used to synthesize the selenium nanoparticles, which were proven to maintain the free radical-scavenging activity of 48.5% [128]. The Lactobacillus casei ATCC393 could be used for biogenic synthesis of selenium nanoparticles under anaerobic conditions. The nanoparticles could have the antioxidant effect with no cytotoxicity at the dose of less than than 25 μg/mL, which is relatively lower compared with other selenium forms [129]. Also, the green synthesis could be used for the fabrication of the selenium nanoparticles with low pollution. The fabrication of the different selenium nanoparticles could offer the feasibility for their application in the industry.

### 4.5. Others

In addition to the inorganic nanoparticles, which could offer antioxidant effects as mentioned above, various other metal oxides with the nanostructures have also been demonstrated to reduce the oxidative damage in recent decades [130]. Gold nanoparticles were used in a wide variety of fields due to their excellent behavior. The Capuli fruit was rich in nutrients and antioxidants, which contains a large variety of total phenolic compounds, flavonoids, tannins, hydroxycinnamic acid, anthocyanins, and sugars. Its extract, CFE, could be applied to synthesize CF-AuNPs by reduction of the Au^3+^. According to this green synthesis method, the formed CF-AuNPs inorganic nanoparticles could exhibit antioxidant by DPPH free radical model and photocatalytic potential. The inhibition % of DPPH for CF-AuNPs was 46.12%, which was due to the binding or adsorption of the Capuli fruit extract phytocompounds on the surface of the formed nanoparticles [64]. The defect-rich lanthanum oxide (La_2_O_3_) nanoparticles were investigated to function as one of the antioxidant activities due to their special electronic configuration of the 4f shielded electrons [131,132]. This inorganic nanoparticle was developed for the purpose of antioxidant activity in human keratinocytes. The results show that this nanostructure could hold the ability to reduce the 30% of free radicals generated by P25 ultraviolet irradiation [133]. Also, the copper oxide nanoparticles (CuO-NPs) was fabricated using the Andean blackberry (*Rubus glaucus Benth.*) fruit and leaf. The antioxidant efficacy of the Andean blackberry fruit CuO-NPs could achieve 89.02%, while 75.92% was achieved for the leaf in scavenging activity of DPPH radical. The antioxidant efficacy was due to the presence of the bioactive molecules from the fruit as well as the leaf extracts on the surface of the formed CuO-NPs [134]. 

## 5. The Safety of the NPs

**Figure 5:**
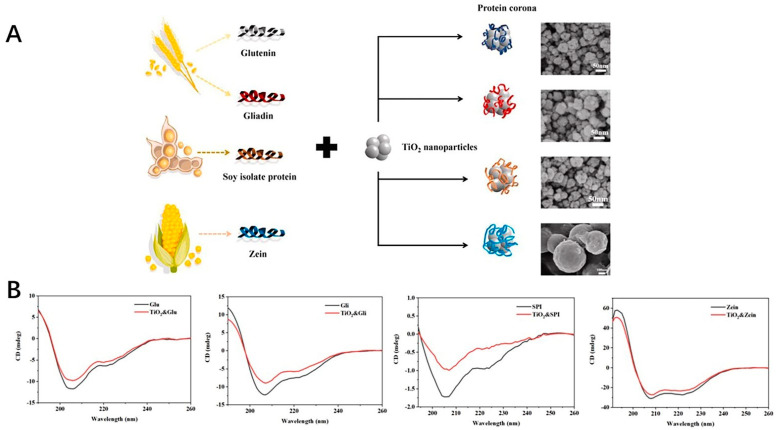
(**A**) The formation of the corona around the TiO_2_ nanoparticles with the protein from foods. (**B**) CD spectra of protein (glutenin, gliadin, soy protein isolate, zein) and protein−nanoparticle coronas.

The safety of the inorganic nanoparticles, which could provide the antioxidant effects, was one of the factors for their application. The research work regarding these nanoparticles focuses on the safety of the application [135,136,137]. The development of the procedure for the safety control still needed to be addressed for the marketing approval. Some of the studies have shown that these inorganic nanoparticles could potentially exhibit some oxide effects to induce the oxide stress [138]. The size and zeta potential, as well as surface and interfacial characteristics should be taken into consideration. These could be associated with the formation of the protein corona, which could affect the behavior of the inorganic nanoparticle in vivo after applied [139,140,141]. One example is that the titanium oxide nanoparticles could form a protein corona around the surface with the protein from foods as shown in Figure 5A. The CD spectra curves of the glutenin, gliadin, soy protein, zein and coronas formed from protein and nanoparticles are shown in Figure 5B. After binding with the nanoparticles, the β-sheet of the glutenin increased slightly, which was also the same as other proteins. It is reported that these nanoparticles could have potential toxicity to the human body. The interaction between titanium nanoparticles and food proteins could form the corona with the thickness of 4–60 nm, which could provide the knowledge for investigating the behavior of these nanoparticles [142]. Also, the research work has shown that the nanoparticles could damage the cells after applied, thus causing the potential toxicity. Several strategies regarding how to reduce the toxicity were developed to fabricate the inorganic nanoparticles [138,143,144].

## 6. Future Perspective

The inorganic nanoparticle could play a role in antioxidant effect, which was potentially to be applied in a wide variety of fields. Although research work involving the mechanism, preparation and decoration of these nanoparticles was demonstrated, the behavior of these nanoparticulates should be well investigated for the purpose of applications. Also, as the metal nanoparticle, their potential toxicity should take into consideration for the safety concern. The technical fabrication, including an eco-friendly method should be investigated, and the machine needs to be well designed to meet the requirement of the broad application. 

## 7. Conclusions

In this work, several of the inorganic nanoparticles which could offer the antioxidant effects were summarized. The recent fabricating method of the inorganic nanoparticle was well introduced. The microfluid could be used for the development of these inorganic nanoparticles, which could act as one method for the industrial continuous production. Also, some biosynthesis and green synthesis methods were used for fabrication of the inorganic metal nanoparticle, which is much more economic, easy to prepare and creates low pollution. Several inorganic nanoparticles, including cerium oxide nanoparticles, Fe oxide nanoparticles, titanium oxide nanoparticles, selenium oxide nanoparticles were developed for the purpose of antioxidant application. Some of these nanoparticles could exhibit antioxidant effects due to the decoration of the bioactivities or fabricated together with the antioxidant moieties. These inorganic nanoparticles could be applied in a wide variety of fields.

## Figures and Tables

**Table 1:** The mechanism of the cerium nanoparticles.

Classification	Types	Mechanism
Antioxidant enzyme-mimetic activities	SOD enzyme mimetic	O_2_^−^ + Ce^4+^ → O_2_ + Ce^3+^ O_2_^−^ + Ce^3+^ + 2H^+^ → H_2_O_2_ + Ce^4+^ [68,91]
	CAT enzyme mimetic	H_2_O_2_ + 2Ce^4+^ + 2OH^−^ → H_2_O + O_2_ + 2Ce^3+^ [68,91]
	phosphatase-like	High catalytic reactivity of erium (IV) nanoparticle, Lewis acidity of the metal with the negatively charged phosphate [70].
	oxidase-like	Oxidize a series of organic substrates without any oxidizing agent [86,87].
	peroxidase-like	The switching ability of Ce^3+^/Ce^4+^, the presence of oxygen vacancies [92]
Antioxidant ROS/RNS scavenging activities	Quenching of hydroxyl radical	Ce_2_O_3_ + 2[OH] → 2CeO_2_ + H_2_O 2CeO_2_ + H_2_O → Ce_2_O_3_ + 1/2O_2_ * [93]
	Quenching of superoxide [94]	
	Quenching of peroxide [95]	
	Quenching of nitric oxide	Ce^4+^ + NO [Ce^4+^ + NO → Ce^3+^ + NO^+^] [93,96]

* In presence of aqueous H^+^.
